# Compensatory role of C3 convertase on the strain difference for C3 protein expression in FVB/N, C3H/HeN and C57BL/6N mice

**DOI:** 10.1186/s42826-020-0036-7

**Published:** 2020-02-18

**Authors:** Ji Won Park, Ji Eun Kim, Mi Ju Kang, Hyeon Jun Choi, Su Ji Bae, Dae Youn Hwang

**Affiliations:** 1 Department of Biomaterials Science, College of Natural Resources and Life Science/Life and Industry Convergence Research Institute, Pusan National University, 1268-50, Samnangjin-ro, Miryang-si, Gyeongsangnam-do South Korea; 2 Laboratory Animals Resources Center, Pusan National University, 1268-50, Samnangjin-ro, Miryang-si, Gyeongsangnam-do South Korea

**Keywords:** C3H/HeN, C57BL/6N, Complement C3, Convertase activity, FVB/N

## Abstract

To investigate the role of complement C3 (C3) convertase on the strain difference for C3 protein expression in three inbred mice strains, we compared the levels of C2, C3 and C4 mRNA, as well as C3 protein and C3 convertase activity in the serum and liver tissue of FVB/N, C3H/HeN and C57BL/6N mice. The level of mRNA, inactive form (InACF) and active form (ACF) for C3 showed a regular pattern, which they were higher in the FVB/N and C57BL/6N mice than C3H/HeN mice. However, the level of C3b fragments (C3bα and β) derived from C3 protein were constantly maintained in the liver of FVB/N, C3H/HeN and C57BL/6N mice in spite of the strain difference on the transcriptional and translation level of C3. Especially, a reverse pattern of the level of mRNA, InACF and ACF for C3 was observed on the activity level of C3 convertase activity. The highest level of C3 convertase activity was measured in C3H/HeN mice, followed by C57BL/6N and FVB/N mice. In case of C3 convertase components, the level of C2 mRNA was higher in C3H/HeN mice than FVB/N and C57BL/6 N mice, while levels of C4 mRNA were higher in FVB/N and C57BL/6N mice than C3H/HeN mice. The current study results provide the first scientific evidence that C3 convertase may play complementary role to overcome the strain difference on the C3 protein expression in FVB/N, C3H/HeN and C57BL/6N mice.

## Introduction

The complement system is a major part of the immune system, present in various organs such as the heart, lung, liver, kidney and gut [1, 2]. This system activates alternative regulation of the complement cascade of three different pathways including the classical, lectin and alternative pathways, subsequently resulting in elimination of the antigenic agent [2]. During these activation processes, C3 plays a central role in promoting opsonic phagocytosis, regulation of humoral immune response, and some T-cell biology [3]. C3 protein can cleavage into C3a and C3b by the proteolytic activity of C3 convertase, which formed with fragments produced in the classical and lectin pathways (C4bC2b, formerly C4b2a) or the alternative pathway (C3bBb) [4]. After cleavage, C3b fragments contribute as one member of the C5 convertase, and subsequentially lead to formation of the membrane attack complex (MAC) consisting of C5b, C6, C7, C8 and polymeric C9 [5].

Few studies have reported effect of strain differences on the complement concentration and its activity, although the complement system has received great attention as one of immune modulators. Several mice strains, including C58, C57BL/6, C57BL/10, DB/1, BALB/c, 129 and C3H/SnHz, exhibit significant levels of hemolytic complement activity corresponding to the role of C5; however, the remarkable enhancement of complement activity has not been measured in some other mice strains due to the absence of C5 [6]. This activity was especially higher in CF-1, B10.D2 and BALB/c stains, but lower in C57B1/10Sh, C57BL10.Br/H2, CBA/J and C3H/J [7]. Significant differences have also been reported in newly established inbred mice strains. These levels were higher in BALB/c, C57BL/6JN, C57BL/10ScN, and B10.D2/Sn (new) strains, as compared to B10.D2/Sn (old) and DBA/2JN [8, 9]. Furthermore, the complement level of serum was measured in 43 mice strains to select a convenient strain having high complement levels. Eight mice strains, including BDP/J, BUB/BnJ, CASA/Rk, and SF/CamEi, showed high complement levels compared to other strains. High levels of C3, C5, C6 and C7 were observed in the BUB mice strain [10]. However, no studies have provided scientific evidence for comparative analysis of the level of C3 convertase-related factors in several inbred strains of mice.

The present study was therefore undertaken to compare the levels of C3 protein and C3b fragment, as well as the levels of C3 convertase activity and its components in the serum and liver of FVB/N, C3H/HeN and C57BL/6N mice.

## Materials and methods

### Animal care and use

The animal protocol used in this study was reviewed and approved by the Pusan National University-Institutional Animal Care and Use Committee (PNU-IACUC; Approval Number PNU-2019-2343). All mice were handled at the Pusan National University-Laboratory Animal Resources Center, which is accredited by the Korea Food and Drug Administration (FDA) (Accredited Unit Number: 000231) and AAALAC International (Accredited Unit Number: 001525). Seven-week-old male FVB/N (*n* = 5), C3H/HeN (n = 5) and C57BL/6N mice (n = 5) were purchased from Koatech Co. Ltd. (Pyeongtaek, Korea). All animals were provided *ad libitum* access to water and a standard irradiated chow diet (Samtako BioKorea Co., Osan, Korea) consisting of moisture (12.5%), crude protein (25.43%), crude fat (6.06%), crude fiber (3.9%), crude ash (5.31%), calcium (1.14%) and phosphorus (0.99%) throughout the experimental period. All animals were maintained in a specific pathogen free (SPF) state under a strict light cycle (lights on at 08:00 h and off at 20:00 h) at 23 ± 2°C and 50 ± 10% relative humidity. After adaptation for 1 week, the mice were euthanized using a chamber filled with CO_2_ gas, and serum and liver samples were subsequently collected for further analysis.

### Quantitative real-time PCR analysis (RT-qPCR)

RT-qPCR was applied to assess the relative quantities of C2, C3 and C4 mRNAs. Total RNA molecules were isolated from frozen liver tissues using RNA Bee solution (Tet-Test Inc., Friendswood, TX, USA). After quantification of the RNA concentration, complement DNA (cDNA) was synthesized using a mixture of oligo-dT primer (Thermo Fisher Scientific Inc., Waltham, MA, USA), dNTP and reverse transcriptase (Superscript II, 18064–014, Thermo Fisher Scientific Inc.). RT-qPCR was then conducted using a cDNA template and 2 × Power SYBR Green (TOYOBO Co., Osaka, Japan), as described in a previous study [11]. The primer sequences used to evaluate the mRNA levels were as follows: C2, sense primer, 5′-CATGG TGGAG AGGAT CTTCA GCTTT GAG-3′, antisense primer, 5′-CAGGT AGTCA TCTCT GTTCT GTTCG ATG-3′; C3, sense primer, 5′-GAACA AACTC ACACA GAGCA AGATC TG-3′, antisense primer, 5′-CAGCT TGGTG ATGTG GTTGC AGCAG TC-3′; C4, sense primer, 5′-CTGGA CAGGA CAGAA CAGTG GAGCA AAC-3′, antisense primer, 5′-CTTCC ACTCT GTTCT TCAGC TGCTT CG-3′; β-actin, sense and antisense primers 5′-TGGAA TCCTG TGGCA TCCAT GAAAC-3′ and 5′-TAAAA CGCAG CTCAG TAACA GTCCG-3′, respectively. The reaction cycle at which PCR products exceeded this fluorescence intensity threshold during the exponential phase of PCR amplification was considered as the threshold cycle (CT).

### Western blot

Total homogenate proteins were extracted from the liver of three inbred mice (FVB/N, C3H/HeN and C57BL/6N mice) using the Pro-Prep Protein Extraction Solution (Intron Biotechnology Inc., Seongnam, Korea). Following centrifugation at 13,000 rpm for 5 min, protein concentrations were determined using a Pierce™ BCA Protein Assay Kit (Thermo Fisher Scientific Inc.). Proteins (30 μg) were then separated by 4–20% sodium dodecyl sulfate-polyacrylamide gel electrophoresis (SDS-PAGE) for 3 h, following which the resolved proteins were transferred to nitrocellulose membranes for 2 h at 40 V. Each membrane was then incubated separately with the following primary antibodies, overnight at 4°C: anti-C3 (Abcam, Cambridge, UK) or anti-β-actin (Sigma-Aldrich Co., St. Louis, MO, USA). The probed membranes were subsequently washed with washing buffer (137 mM NaCl, 2.7 mM KCl, 10 mM Na_2_HPO_4_, 2 mM KH_2_PO_4_, and 0.05% Tween 20), followed by incubation with horseradish peroxidase-conjugated goat anti-rabbit IgG (1:1000 dilution) (Zymed Laboratories, South San Francisco, CA, USA) at room temperature for 2 h. Finally, the blots were developed using a Chemiluminescence Reagent Plus kit (Pfizer Inc., Gladstone, NJ, USA). The signal band image for each protein was acquired using a digital camera (1.92 MP resolution) of the FluorChem® FC2 Imaging system (Alpha Innotech Corporation, San Leandro, CA, USA). Protein densities were semi-quantified using the AlphaView Program version 3.2.2 (Cell Biosciences Inc., Santa Clara, CA, USA).

### ELISA for C3 convertase activity

The C3 convertase activity was determined using the C3 convertase ELISA Kit (MyBiosource, San Diego, CA, USA), in accordance with the manufacturer’s protocols. Serum from each mouse was subsequently placed in individual wells of a precoated antibody plate, and incubated at 37°C for 90 min, followed by addition of 100 μL biotinylated mouse C3c antibody to each well, and further incubation at 37°C for 60 min. Subsequently, 100 μL of enzyme-conjugate liquid was added to each individual well, and incubated at 37°C for 30 min. Finally, 100 μL of the Color reagent liquid and Color reagent C was added to each well, after which the color alteration was determined by using a Vmax plate reader (Molecular Devices, Sunnyvale, CA, USA) at 450 nm. The concentration of C3c (ng/mL) was determined by standard curve and used to represent C3 convertase activity.

### ELISA for C3 protein concentration

Concentration of C3 protein in the serum was measured using the mouse complement C3 ELISA kit (Abcam, ab157711) according to the manufacturer’s protocols. Briefly, serum of each mouse was diluted 1:75,000 and pipetted into designated wells of kit. The plate was incubated for 20 min at room temperature, washed, and a 1× enzyme-antibody conjugate was added. After incubation for 20 min at room temperature and additional washing, TMB substrate was added and the color alteration was determined by using a Vmax plate reader (Molecular Devices) at 450 nm.

### Statistical analysis

Statistical significance was evaluated using one-way analysis of variance (ANOVA) (SPSS for Windows, Release 10.10, Standard Version, Chicago, IL, USA) followed by Tukey’s post hoc t-test for multiple comparisons. Data are presented as mean ± SD (standard deviation). *p* < 0.05 is considered to indicate a statistically significant difference.

## Results

### Strain difference on the transcriptional and translational regulation for C3 proteins

**Fig. 1:**
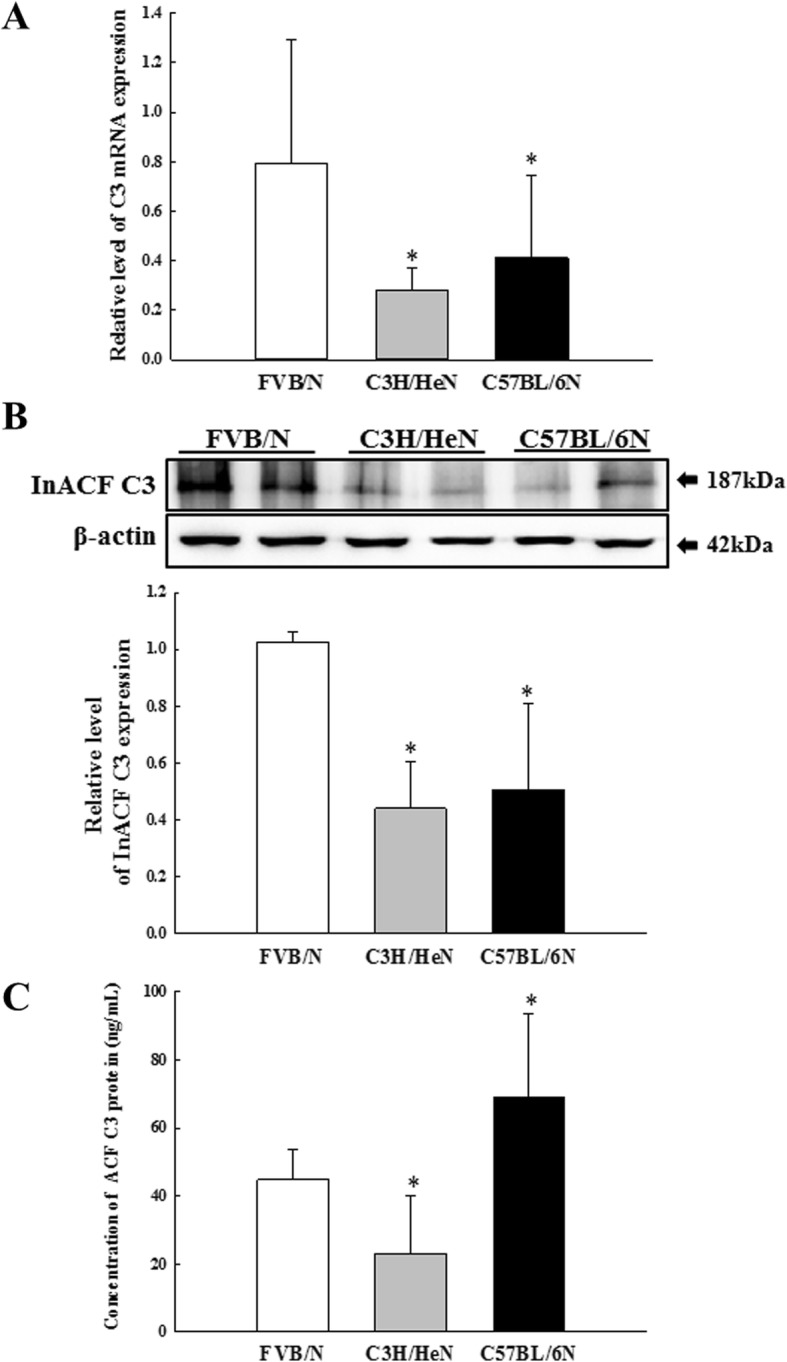
Expression level of mRNA, InACF and ACF for C3. **a** The levels of C3 transcripts in the total mRNA of liver tissue were measured by quantitative real-time (qRT)-PCR analyses using specific primers. The mRNA level of each gene was calculated based on the intensity of actin as an endogenous control. **b** Expression levels of InACF C3 proteins was determined by Western blot analysis using HRP-labeled anti-rabbit IgG antibody. Band intensities were determined using an imaging densitometer, and the protein expressions were calculated relative to the intensity of β-actin. **c** The concentration of ACF C3 was measured using the serum of three inbred mice using the Mouse Complement C3 ELISA Kit. This assay detects as low as 1.658 ng/mL. Two to three mice per group were used for the preparation of the tissue lysates and serum, and the PCR, Western blot analysis and ELISA were assayed in duplicate for each sample. Data are reported as the mean ± SD. * indicates *p* < 0.05 compared to the FVB/N group. Abbreviation: InACF C3, Inactive form C3; ACF C3, Active form C3

To analyze the strain difference on the transcriptional and translational regulation for C3 proteins in three inbred mice, we measured the expression levels of mRNA, InACF and ACF for C3 in the liver homogenates and serum of FVB/N, C3H/HeN and C57BL/6N mice. The expression levels of mRNA, InACF and ACF for C3 showed a similar pattern in three inbred strains; levels were lower in the C3H/HeN mice than FVB/N and C57BL/6N mice, although the decrease rate varied for each analysis. The highest level of mRNA and InACF for C3 was observed in FVB/N mice, while those of ACF C3 protein detected in C57BL/6 Nmice (Fig. 1a, b and c). These results firstly suggest that the significant strain difference on the transcriptional and translational regulation of C3 protein may determine in FVB/N, C3H/HeN and C57BL/6N mice.

### Strain difference on the production of C3b fragment derived from C3 protein

**Fig. 2:**
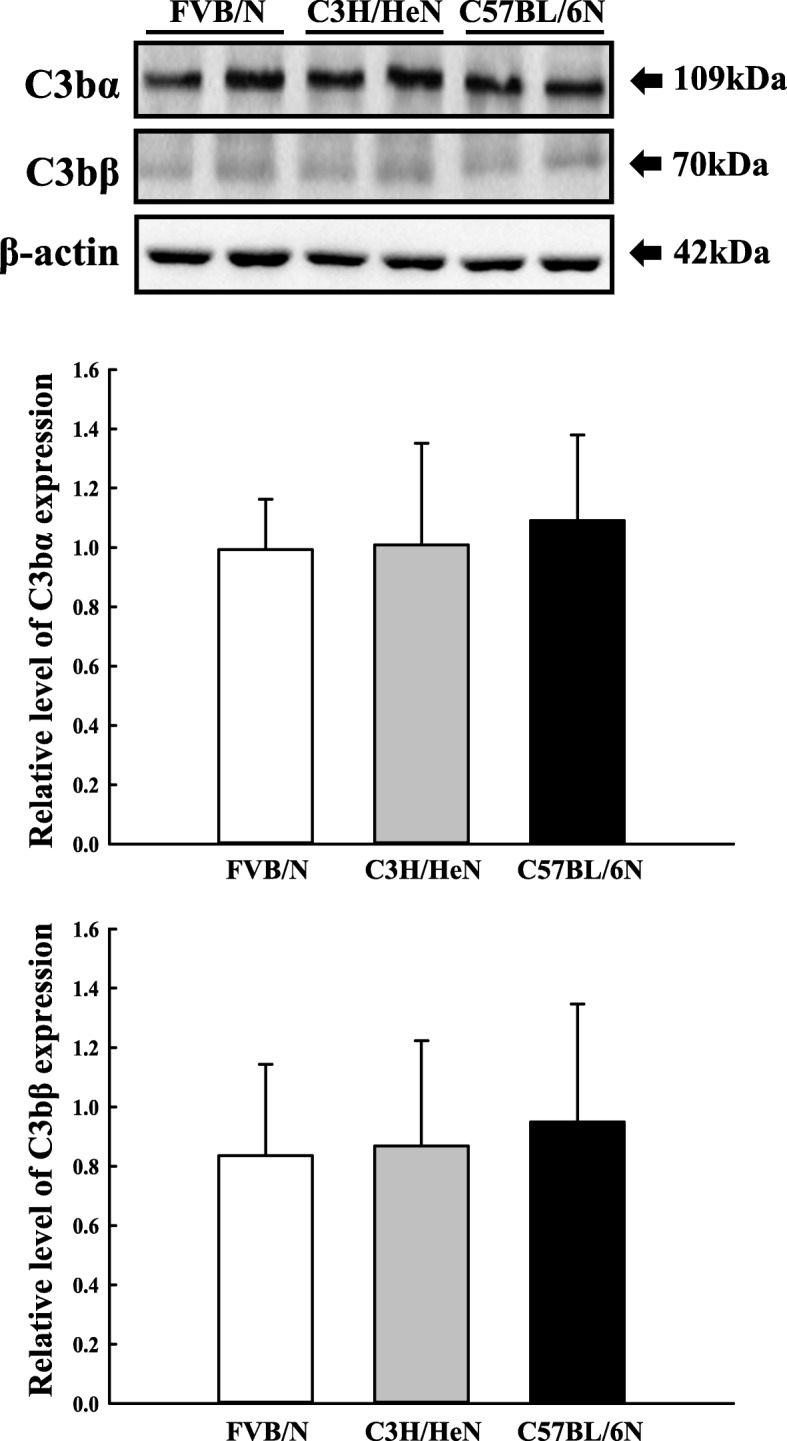
Level expression of C3b fragment. Expression levels of C3bα and C3bβ fragment were determined by Western blot analysis using HRP-labeled anti-rabbit IgG antibody. Band intensities were determined using an imaging densitometer, and the expressions of the protein were calculated relative to the intensity of β-actin. Two to three mice per group were used for the preparation of the protein homogenates, and Western blot analysis were assayed in duplicate for each sample. Data are reported as the mean ± SD. *p < 0.05 as compared to the FVB/N group

We investigated whether the strain difference on the transcriptional and translational regulation of C3 could be reflected in the production of C3b fragment derived from C3 protein. To achieve these, the expression level of C3bα and β protein was measured in the liver homogenate of three inbred mice using Western blot analysis. We observed that the expression level of these two proteins were maintained constant in the liver tissue of FVB/N, C3H/HeN and C57BL/6N mice (Fig. 2). Especially, the expression level of C3bα fragment were higher than those of C3bβ fragment in three inbred mice regardless of strain (Fig. 2). The above results showed that the production of C3 fragment derived from C3 protein may not reflected in the strain difference on the transcriptional and translational regulation of C3 in three inbred mice. Furthermore, these results indicate that three inbred mice were maintained constant level of C3b fragment, one of key component for C5 convertase, regardless any significant strain difference on the upstream process of complement pathway.

### Compensatory regulation of C3 convertase against strain difference on the expression level of C3 protein

**Fig. 3:**
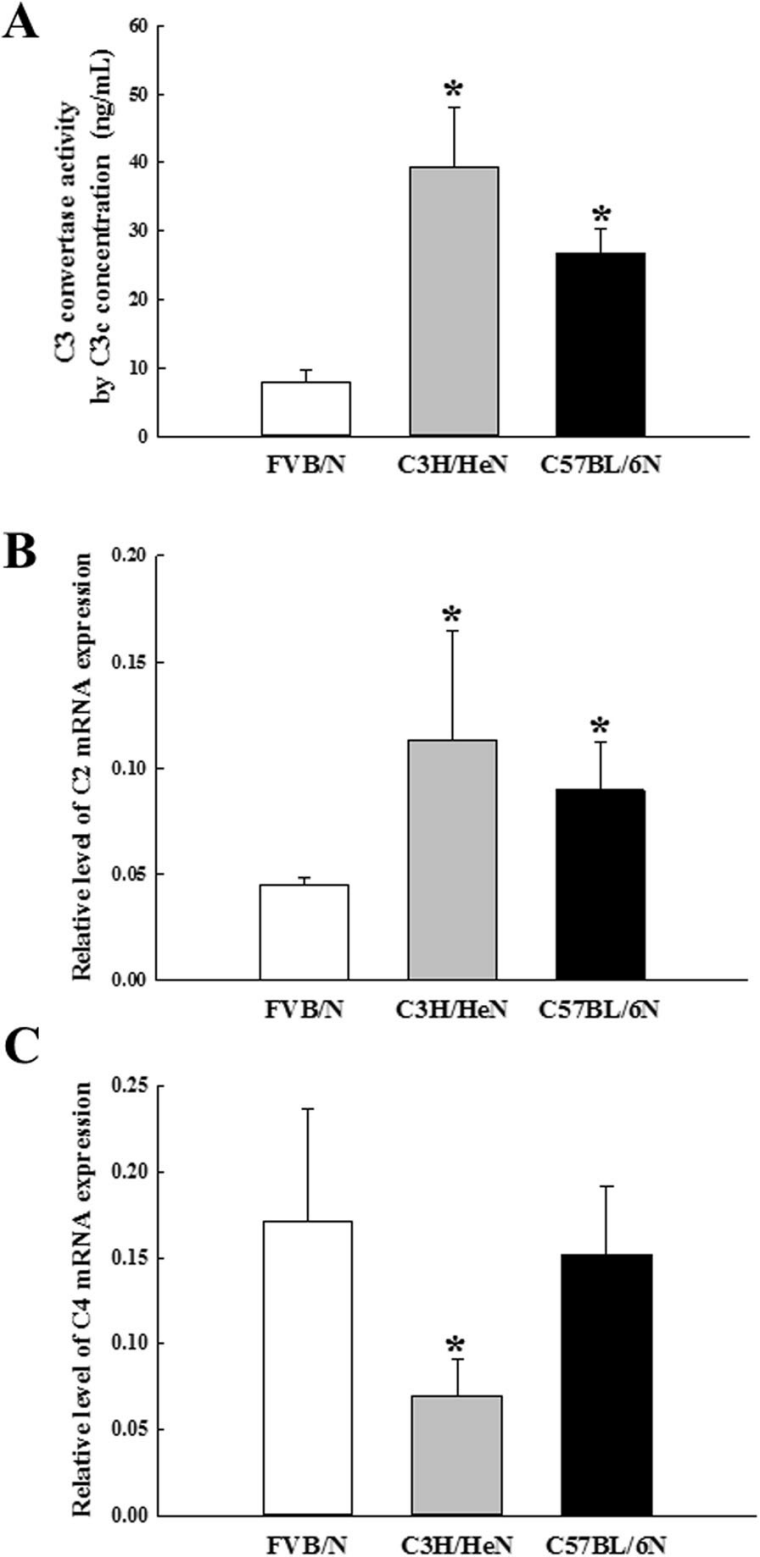
Level of C3 convertase activity and C2/C4 mRNA. **a** C3 convertase activity was measured using the serum collected from three inbred mice using a Mouse Complement C3 convertase (C3c) ELISA Kit. This assay can detect levels as low as 0.5 ng/mL. **b** and **c** The levels of C2 and C4 transcripts in the total mRNA of liver tissue were measured by quantitative real-time (qRT)-PCR analyses using specific primers. The mRNA level of each gene was calculated based on the intensity of β-actin as an endogenous control. Two to three mice per group were used for the preparation of tissue lysates and serum, and the PCR and ELISA analysis were assayed in duplicate for each sample. Data are reported as the mean ± SD. * indicates *p* < 0.05 when compared to the FVB/N group

To investigate the possible role of C3 convertase on the inconsistency between the levels of C3 protein and the production of C3b fragment derived from C3 protein, the level of C3 convertase activity and C2 and C4 mRNA expressions were measured in the serum and liver tissue of FVB/N, C3H/HeN and C57BL/6N mice. The levels of C3 convertase activity were higher in C3H/HeN and C57BL/6N mice than FVB/N mice, although the increase rate was greater in C3H/HeN than C57BL/6N. These patterns in three inbred mice was the exact opposite of those of C3 proteins (Fig. 3a). A similar pattern was observed on the expression pattern of C2 mRNA. This mRNA was remarkably enhanced in C3H/HeN and C57BL/6N mice compared with FVB/N mice (Fig. 3b). However, a different pattern was observed on the expression level of C4 mRNA. This level was significantly decreased in C3H/HeN mice compared with FVB/N and C57BL/6N mice (Fig. 3c). These results, therefore, determined that strain difference on the C3 convertase activity and it components level can contribute the compensatory adjustment for strain difference on the expression level of C3 protein in the three inbred strains.

## Discussion

Inbred mice have long been used in various immunological researches, including cell-mediated immunity, allergic response and anti-tumor mechanism, due to their reliability, reproducibility and scope of improvement of animal-based experiments [12]. As immunological researches involving inbred strains increases, strain differences of mice on the immunological response has received growing attention [13]. As part of this effort, we investigated the strain difference on the C3 convertase-related factors among the major components of the complement system in three inbred mice. The results of the present study provide the first evidence that C3 convertase may compensate strain difference on the transcriptional and translation regulation of C3 to maintain the homeostatic complement response in FVB/N, C3H/HeN and C57BL/6N mice. Furthermore, these results show that the strain difference on the level of C3 convertase-related factors can be considered as one of the important factors during immunological studies. However, our study has been carried out on limited analyses of C3 convertase-related factors and only three inbred mice.

Among the several complements, C3 maintains a high concentration (1.2–1.5 mg/mL) in serum and body fluids. Most of the serum C3 is produced from liver tissue, but lesser levels are locally secreted from various cell types, including lymphocytes and macrophages [3, 14]. In this study, we selected the liver tissue as the major target organ to analyze the levels of C3 convertase-related factors. The levels of C2, C3 and C4 mRNA were successfully measured in the liver tissue of three inbred mice, while C3 convertase activity and ACF C3 concentration were detected in the serum of the same mice. These results provide additional evidence that the liver tissue can be considered as the target organ for C3 convertase-related factors, although further studies are required to measure smaller fragments such as C2b, C3a and C4a derived from the cascade pathway.

It is impossible to make a clear comparison with previous studies since no paper has applied similar methods as the present study. However, a few studies provide clues to analyze the complement activity of few inbred mice used in this study. The hemolytic complement activity was detected as positive in C57BL/6J and C3H/SnHz mice [6]. This level was lower in C3H/J than CF-1 and BALB/c [7]. Serum from C57BL/6JN showed about 1.3 C’H_50_/mL of complement activity and was 4.2 times lower than BALB/cAnN [9]. However, the complement activity was not analyzed in FVB/N mice. In the current study, we have focused the C3 convertase activity as one of key factors during classical and lectin pathway for complement activation. The activity of C3 convertase in the serum was detected as height level in C3H/HeN mice when compared with other strains. However, these results for the C3 convertase activity cannot be directly compared to previous results for complement activity because there is a difference in the strain of mice analyzed.

Meanwhile, a significant strain difference in mice has previously been reported for levels of C5, the main hemolytic complement [13]. A loss-of-function mutation in the C5 gene was detected in several mice strains such as A/J, AKR/J, DBA/2, DA/1, FVB/NJ and SWAR. This mutation induces an alteration in pathogen susceptibility against *Bacillus anthracis* and *Candida albicans*, which was found to be decreased in the above strains as compared to C57BL/6 strain [11]. But, strain difference on the level and gene mutation of C3 was not reported in any previous studies. In this study, we compared only mRNA, InACF and ACF of C3 in three inbred strain. The similar pattern for strain difference was observed at transcriptional and translation level for C3 gene. However, FVB/N strain was commonly used in the recent study and previous studies although target gene was different in each experiment. Actually, it is not known whether C3 convertase activity is associated with C5 mutation in the FVB/NJ strain. A further study is required to determine whether decrease of C3 convertase activity is accompanied with functional deficiency of C5.

In addition, the results of the present study show that the level of C3b fragment derived from C3 protein was consistently maintained in all three inbred mice, although significant strain difference on the transcriptional and translational regulation of C3 protein. The cleavage process of C3 fragment were complemented by the differential regulation of C3 convertase activity in in three inbred mice. The FVB mice with high level of C3 protein showed the relatively low level of C3 convertase activity, the C3H/HeN mice with low level of C3 protein exhibited the high level of C3 convertase activity as show Figs. 1 and 3. These results indicate that strain difference between C3 protein expression and C3 convertase activity is in a complementary relationship. Above regulation can be contributed the same potential to response for complement-mediated responses including target cell lysis, opsonization, inflammation and immune clearance in FVB/N, C3H/HeN and C57BL/6N mice [15]. The consistency of C3 convertase-derived C3b fragment can attributed to the homeostatic regulation of the immune system [10]. However, further analysis of the exact cause and molecular mechanism for this phenomenon is essential in various mice strain.

Meanwhile, our study has a limitations on the analysis method for C2 and C4 as main component of member C3 convertase [16]. In the present study, qRT-PCR analysis was performed to measure the mRNA level of C2 and C4 in the liver tissue of three inbred strains as shown Fig. 3. This method has been selected based on the several studies that evaluated the mRNA level of C2 and C4 to study the mechanism on the regulation of two genes. The C2 and C4 mRNA expression were quantified with qRT-PCR in IFN-γ stimulated mononuclear cells [17]. A significant alterations on the mRNA content of C2 and C4 in the liver, lung, heart and intestine was detected between MRL (lpr/lpr) strain and MRL (+/+) strain although there are no difference in the spleen [18]. Also, the increase of mRNA stability for C4 gene expression was reported as major mechanism of IFN-γ stimulated HepG2 cells [19]. However, further study are needed to compare the exact protein level of C3 convertase components in three inbred strains.

## Conclusions

**Fig. 4:**
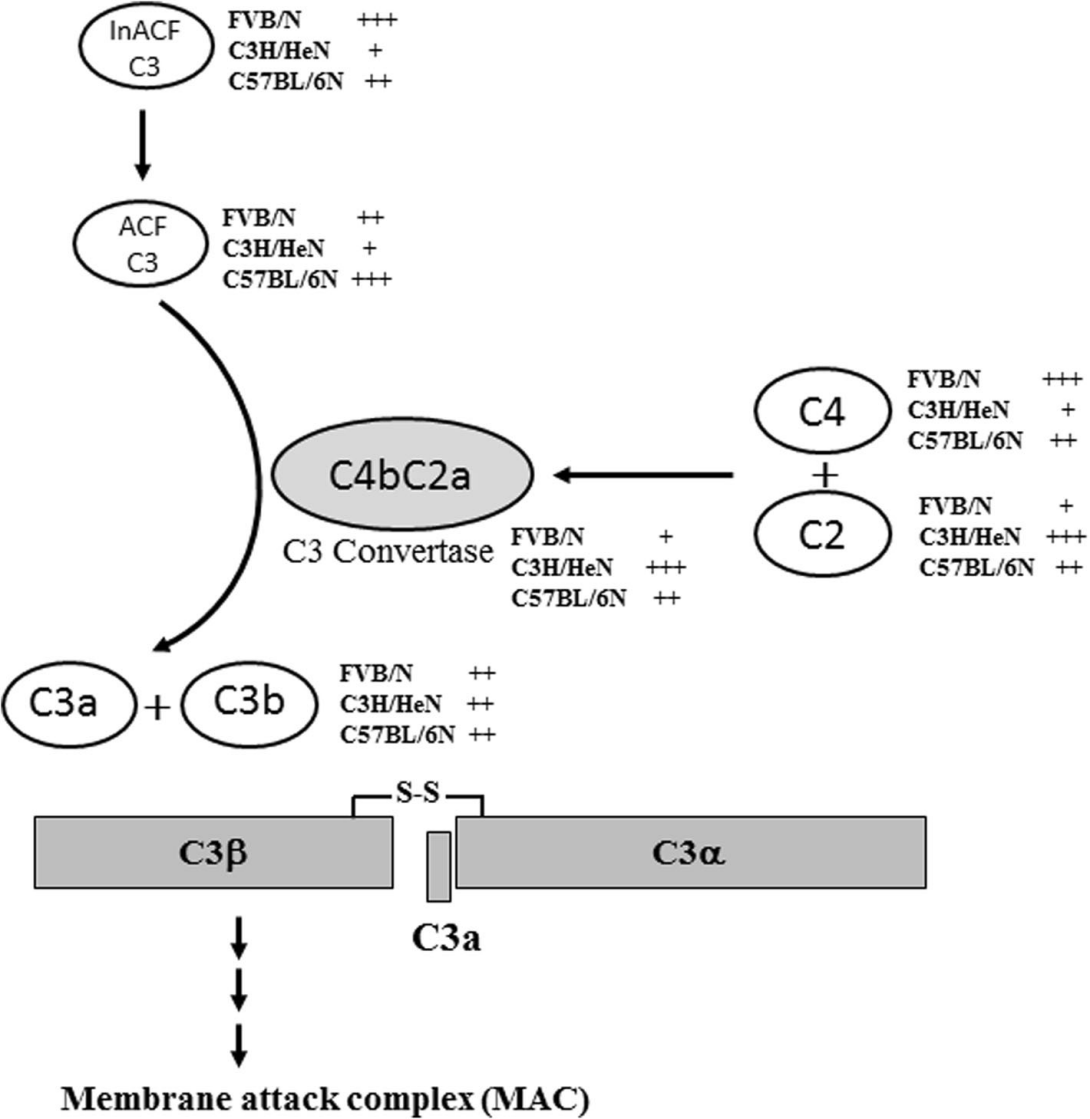
Summary for compensatory role of C3 convertase on the cleavage pathway of C3 protein. C3 convertase, comprising the C4b and C2b fragments, leads to the production of C3a and C3b. Additional deposited C3b forms the C5 convertase, which reacts with other complement factors to form membrane attack complex (MAC). Abbreviations: +, low level of expression; ++, medium level of expression; +++, high level of expression

We investigated the strain difference for levels of C3 convertase-related factors in the serum and liver of three inbred mice, namely, FVB/N, C3H/HeN and C57BL/6N mice. Taken together, our results indicate that C3 convertase activity can play a complementary role to overcome the strain difference on the transcriptional and translational regulation of C3 and maintain the constant level of C3b fragment in FVB/N, C3H/HeN and C57BL/6N mice (Fig. 4). Furthermore, these results suggest that any immunological study using FVB/N, C3H/HeN and C57BL/6N mice should consider the strain difference and consistence on the levels of C3 convertase-related factors.

## Data Availability

Available.

## Abbreviations

ACF: Active form
C3: Complement C3
CT: Threshold cycle
FDA: Food and Drug Administration
InACF: Inactive form
MAC: Membrane attack complex
PNU-IACUC: Pusan National University-Institutional Animal Care and Use Committee
SDS-PAGE: Sodium dodecyl sulfate-polyacrylamide gel electrophoresis
